# Why, how, and whether to assess digital health literacy

**DOI:** 10.1177/20552076261452376

**Published:** 2026-05-14

**Authors:** Mika Uitto

**Affiliations:** 1 Seinäjoki University of Applied Sciences, Seinäjoki, Finland

**Keywords:** digital health literacy, ehealth literacy, assessment, digital health < general, digital transformation

## 1. Introduction

The digital transformation and increasing reliance on digital health technologies has brought the term digital health literacy (dHL) – ‘*a set of knowledge and skills that enable citizens to promote and maintain good health and wellbeing through digital technology’*^1^ – to the attention of many researchers around the world. However, to date, a substantial body of research on dHL has focused on the development and validation of assessment tools and on categorising individuals according to their knowledge and skills. While the quantification of dHL has its value, it also risks victim blaming and oversimplifying the concept’s complex, evolving, and context-dependent nature.

Before choosing to do assessments or developing new tools, researchers should explore *why* dHL should be assessed, *how* it can be done and *whether* – or to what extent – it is relevant. The European Digital Health Literacy Conference was held in Aalborg, Denmark in November 2025. One of its workshops, titled ‘*How, why and whether to assess digital health literacy’*, brought together 31 participants, including researchers, students, healthcare professionals, and other stakeholders, from eight countries across Europe. This paper draws its inspiration from this workshop.

## 2. Why

The first question of *why dHL should be assessed* is arguably the least controversial of the three. The rationale for assessing dHL mirrors that of health literacy (HL): identifying individuals in vulnerable situations to better target support and actions.^2–4^ Knowing who struggles and why is crucial for guiding interventions (e.g. recognising communication needs or digital service design needs), supporting informed decision making (e.g. prioritising resources, developing equitable policies) and enabling evaluation (e.g. whether interventions are effective or not).^2,4^ dHL has been described as a ‘*super determinant*’ of health.^5^ Limited dHL can make it difficult for people to judge the reliability of online health information, increasing the risk of misunderstandings and misinformation, and can hinder their ability to use digital tools for timely guidance or participate in co-designing health services.^6^

## 3. How

The second question of *how can dHL be assessed* is more complex. Recent scoping and systematic reviews,^7–9^ supplemented by targeted searches, identify more than 20 tools designed to assess individuals’ dHL. These tools vary in many ways.

### 3.1. Evolving social construct

One reason for this variation is that the tools are based on different theoretical backgrounds and definitions. dHL is a social construct; a status that Pleasant^10^ describes as a double-edged sword. On the one hand, it may help to prevent concepts from ‘*fragmenting under stress*’, but on the other, it may ‘*prevent the field from building a defined, valid, objective, and reliable evidence base that can produce systematic reviews identifying best practices based on a high strength of evidence*’.^10^

Furthermore, dHL is not static. Norman & Skinner^11^ have argued that ‘*it is a process-oriented skill that evolves over time as new technologies are introduced and the personal, social and environmental contexts change’*. To illustrate the variation and evolution within dHL, early tools such as eHEALS^11^ were grounded in definitions and models that focused primarily on the comprehension of online health information. Subsequent work has expanded this understanding, for example to include online interaction,^12,13^ online security and privacy, use of digital health services,^13,14^ and even self-tracking and self-managing.^15^

More recent research suggests that emerging technologies like AI, health monitoring devices, and remote services are likely to introduce additional layers of competency requirements for citizens in the future.^16^ Consequently, dHL should be understood as a multidimensional and evolving concept that may hold different meanings for different people, which in turn results in diverse assessment tools measuring different aspects of the construct.

### 3.2. Self-assessment vs. performance-based items

At a practical level, there are two main types of approaches for assessing dHL: Subjective (self-assessment) and objective (performance-based).^17^ Subjective measures typically require respondents to evaluate their own perceived competencies against specific prompts. For example, ‘*When you search online for information on health, how easy or difficult it is for you to judge whether the information is reliable*?’, *1 = very difficult, 4 = very easy*.^12^ The main advantages of subjective measures are that they are easy to administer, suitable for rapid deployment, and associated with a lower risk of stigma compared with objective assessments.^17^ These characteristics make them particularly attractive for population-level surveys. However, a key limitation of subjective items is the difficulty in determining whether responses reflect actual skill levels or merely self-efficacy.^10^ Certain groups may also be more likely to systematically over- or under-estimate their competencies.^17^ For example, men tend to overestimate themselves, while women underestimate.^18^

In contrast, objective (performance-based) items require respondents to demonstrate a skill or apply knowledge rather than provide a self-assessment. For instance, respondents may be shown an image of a digital service interface and asked to answer a question such as ‘*What kind of information do you expect to find when clicking on button A*?’.^13^ Performance-based items may be particularly useful when a verifiable estimate of an individual’s actual abilities is required, rather than a perceived one.^17^ Nevertheless, a significant challenge is that respondents are aware that their skills are being evaluated. This may increase the risk of stigma and shame, especially in clinical settings, if their responses suggest limitations in their abilities.^2,17^ Moreover, performance-based assessments often assume the existence of universally applicable competencies that can be objectively measured and are relevant to individuals across international contexts. Where such competencies are proposed, it is important to consider whether they truly reflect dHL or merely functional literacy.

### 3.3. The ‘best’ tool

Given the wide range of available tools and approaches for assessing dHL, the question arises as to how researchers can identify the ‘*best’* tool. The short answer is that they cannot, as no single tool is superior for all contexts. Although the absence of a gold standard in dHL assessment has been recognised as a critical limitation in the field,^19^ more recent work has increasingly embraced the view that there is no single universal ‘*best’* tool, but rather tools designed for different needs and purposes.^3^ For example, even eHEALS^11^ – often criticized for being outdated in contemporary settings^3,19,20^ – is not inherently problematic when it is used to assess what it was designed to measure: individuals’ perceived ability to find, understand, and evaluate health information from relatively static, Web 1.0–based internet sources and to use this information to address health problems. The best tool is the one that fits the purpose of the research.

Selecting an assessment tool, therefore, may depend on several contextual and practical factors. These can include the purpose of the assessment, sample size, and the tool’s length, quality, and content. Cultural and linguistic validation is also critical to ensure accuracy and relevance. Resource constraints may force researchers to compromise and select a tool that is already available instead of creating or validating the ideal tool for their purpose. Not to mention, that especially early-career researchers might find their choices subtly steered by their mentors, whose guidance tends to align with their own research agenda.

## 4. Whether

While questions of why and how to assess dHL are important, it is perhaps equally crucial to critically examine *whether* – or to what extent – *it is relevant to assess dHL*. By recognising the challenges and limitations involved, researchers can better determine whether they should allocate their resources to assessing individuals’ dHL levels or concentrate on other priorities.

### 4.1. What are adequate levels of dHL?

Research on dHL often reports findings like ‘*X% of citizens have low dHL’*. Such statements raise questions not only about how dHL is operationalised in these studies but also about how categories such as ‘*low’*, ‘*limited*’, or ‘*inadequate*’ are interpreted. Many assessment tools for dHL divide respondents into such labels using cut-offs set by researchers using quantitative methods. While this may make it easier to interpret the findings and give meaning to mere numerical values, it risks reinforcing the idea that there are universal thresholds for what constitutes ‘*enough’* dHL. In practice, the adequacy of an individual’s dHL is context-dependent and depends on how difficult or easy the websites, services, or other solutions are to find, understand and use for citizens.^2^ The ideal situation is when a citizen’s skills align with the system’s demands and complexity.^2^ If websites, services and solutions are accessible and easy to use, even lower levels of dHL may be enough.

This terminology can also be interpreted as implying that the individual is the core problem. Reducing people to numbers or deficit-based labels may lead to victim blaming and misinterpretations that place responsibility on the individual. This shifts attention away from the diverse contexts, lived realities, and structural determinants of health that shape inequalities.^21^ To mitigate deficit-based narratives that focus on what people ‘*can’t do or don’t have’*, and that contribute to stigmatisation and stereotypes, some researchers have suggested shifting from assessment approaches that identify limitations to those that emphasise individuals’ strengths.^22^

### 4.2. There are other ways of contributing to dHL than assessing individuals’ levels

Understanding the interplay between individuals’ abilities and system demands encourages us to examine dHL gaps not only on the individual level, but also in relation to the broader factors that influence them. Blaming people for lacking abilities defined by others as adequate is neither an accurate approach nor a true reflection of the concept.^10^ Weiss^23^ has argued: ‘… *we need to stop performing studies to demonstrate things we already know—that patient education material is often too difficult to understand, or that many patients have limited literacy skills. Instead, we need more high-quality HL and communication research that focuses on meaningful patient-oriented outcomes*’.

Although research has advanced areas such as organisational HL – aiming to make it easier for citizens to find, understand and use health information and services^24^ – similar research with a dHL focus is only beginning to emerge.^25^ Moreover, while strengthening organisational HL is crucial, it represents only a partial solution for achieving long-term sustainability at a system level.^26^ Actions are also needed across political, technological, and commercial levels.^27^

In the workshop, participants were invited to reflect on the question: ‘*What do you find interesting in dHL research beyond assessing individuals’ levels?*’. The participants’ responses and the discussion that followed clustered around the following areas:

*i) Organisational and professional dHL*: focusing on integrating dHL within healthcare organisations and professionals’ practices to remove barriers for dHL.
*ii) Transforming knowledge into action*: applying the current knowledge on dHL to designing effective interventions and user-friendly technologies.
*iii) Education and training*: supporting both healthcare professionals and citizens in interacting with digital tools and solutions.
*iv) Acknowledging the system-level*: exploring how individuals’ dHL interacts with meso- and macro-level factors.
*v) Preparing for new technologies and future changes*: studying the impact of advancing technologies like AI on dHL.

## 5. Conclusion

*Why* assessing dHL is important lies in the need to understand who struggles with digital health and why. Understanding of people in vulnerable situations should directly feed into processes to support informed decision making and creating inclusive digital health platforms, services, and interventions that mitigate known challenges, rather than just categorising or labelling individuals. If assessment does not lead to concrete action to improve health, wellbeing, or decrease inequalities, it risks ending up serving little purpose beyond the assessment itself.

*How* it can be assessed is challenging, as it is a multi-dimensional and constantly evolving concept that can be understood in various ways. No single assessment tool is universally ‘*best’* for evaluating dHL. Researchers must acknowledge this and understand, what it is that they specifically want to assess, and make their decisions accordingly. This helps ensure that the tool selection is appropriate for their needs and the results are interpretable within the relevant context.

*Whether* it is relevant to assess dHL requires critical consideration. Researchers should reflect on whether they wish to approach dHL challenges as a personal deficit or as a system-level issue, and whether the assessment is likely to generate genuinely new insights beyond what is already known. Assessing individuals’ dHL can be valuable, but there are also many other ways to contribute to the research. Based on the workshop reflections, future research could focus on what healthcare organisations and professionals can do to make it easier for citizens to find, understand, and use digital health solutions, applying the knowledge we already have into action, supporting education and training, exploring how system factors influence dHL and health inequality, and preparing for new technologies and future changes.

To summarise, assessing individuals is not the only way to contribute to dHL research, but it can be relevant when it provides new insights about people in vulnerable situations, uses appropriate tools, is carefully interpreted within context, and leads to actions that genuinely promote health and wellbeing.
